# Indoor air quality of everyday use spaces dedicated to specific purposes—a review

**DOI:** 10.1007/s11356-017-0839-8

**Published:** 2017-11-30

**Authors:** Mariusz Marć, Monika Śmiełowska, Jacek Namieśnik, Bożena Zabiegała

**Affiliations:** 1 Department of Analytical and Ecological Chemistry, Faculty of Chemistry, Opole University, Opole, Poland; 2 Department of Analytical Chemistry, Gdańsk University of Technology, Narutowicza Str. 11/12, 80-233 Gdańsk, PL Poland

**Keywords:** Indoor environment quality, Cooking places, Residential rooms, Basements, Residential garages

## Abstract

According to literature data, some of the main factors which significantly affect the quality of the indoor environment in residential households or apartments are human activities such as cooking, smoking, cleaning, and indoor exercising. The paper presents a literature overview related to air quality in everyday use spaces dedicated to specific purposes which are integral parts of residential buildings, such as kitchens, basements, and individual garages. Some aspects of air quality in large-scale car parks, as a specific type of indoor environment, are also discussed. All those areas are characterized by relatively short time use. On the other hand, high and very high concentration levels of xenobiotics can be observed, resulting in higher exposure risk. The main compounds or group of chemical compounds are presented and discussed. The main factors influencing the type and amount of chemical pollutants present in the air of such areas are indicated.

## Introduction

According to the information published in the scientific literature and the experts from the National Human Activity Pattern Survey (NHAPS) of the USA, the average adult male spends about 87% of his daily time in enclosed spaces (defined as an indoor environment) and approximately 6% in various types of vehicles (private cars or public transport) (Klepeis et al. 2001; Hollbacher et al. 2017). Due to the long exposure time of a human in various types of indoor areas/enclosed spaces (especially in domestic areas), it is justified to conduct a wide range of research aimed at obtaining precise analytical information on indoor air quality (defined by the type and the amount of the chemical compounds present in it). The modern philosophy of designing and building/constructing residential buildings and apartments is mainly focused on creating indoor areas, which in daily use minimize the consumption of electricity and thermal energy due to a very good thermal insulation of the building. Currently, residential areas are characterized by very good tightness (e.g., by using new types of tight windows) and central or individual ventilation systems installed inside the building or apartment (Weschler 2009; Kauneliene et al. 2016; Stazi et al. 2017). The high tightness of modern residential areas forces the designers and constructors of buildings to constantly improve the efficiency of the ventilation system. The long exposure time of a human in tight residential areas and apartments, very often the lack of sufficient air exchange and ventilation system malfunction, inhabitant activities, and their lifestyle (individual for each user of indoor areas) related to daily activities in the apartments, significantly affect the quality of the indoor environment (Weschler 2009; Kauneliene et al. 2016). The effect of these factors is that the level of chemical compounds in the indoor environment is much higher than in ambient (atmospheric) air (Guo et al. 2003). Additionally, the quality of indoor environment is also influenced by the presence of pollutants dispersed in the ambient air as a result of the intense movement of air masses or precipitation and physicochemical changes that occur in the presence of solar radiation and oxidative agents, like tropospheric ozone, nitrogen oxides, and hydroxyl radicals (Sillman 1999; Atkinson 2000; Słomińska et al. 2014; Masih et al. 2017).

According to literature data, there are three primary factors that significantly affected the quality of the indoor environment in residential areas: (i) human activity in indoor environment; (ii) building and constructing materials, furniture, and various types of indoor equipment; and (iii) outdoor air quality, including outdoor chemical compound emission sources. Mainly, the type and quantity of the xenobiotics present in indoor air is affected by various forms of human activity in residential areas (Schlink et al. 2016). The intensity of the impact of these factors on indoor air quality is mainly determined by the socioeconomic status of the inhabitants and the intensity and type of performed renovations and restoration actions (Hameed et al. 2004; D’Souza et al. 2009). The main chemical compounds present in the indoor environment having an important impact on its air quality, and the well-being and mental and physical conditions of the occupants of a given residential area in the long term, (Suryawanshi et al. 2016) are polycyclic aromatic hydrocarbons (PAHs) (Ma and S. Harrad 2015; Chen et al. 2017b); benzene, toluene, ethylbenzene, and xylene (BTEX) compounds as representatives of the volatile organic compounds (VOCs) (Hazrati et al. 2016; Masih et al. 2017); formaldehyde and acetaldehyde (Katsoyiannis et al. 2008; Plaisance et al. 2013; Jiang et al. 2017); and terpenes, e.g., α-pinene, 3-carene, or d-limonene (Curci et al. 2009; Król et al. 2014).

**Fig. 1:**
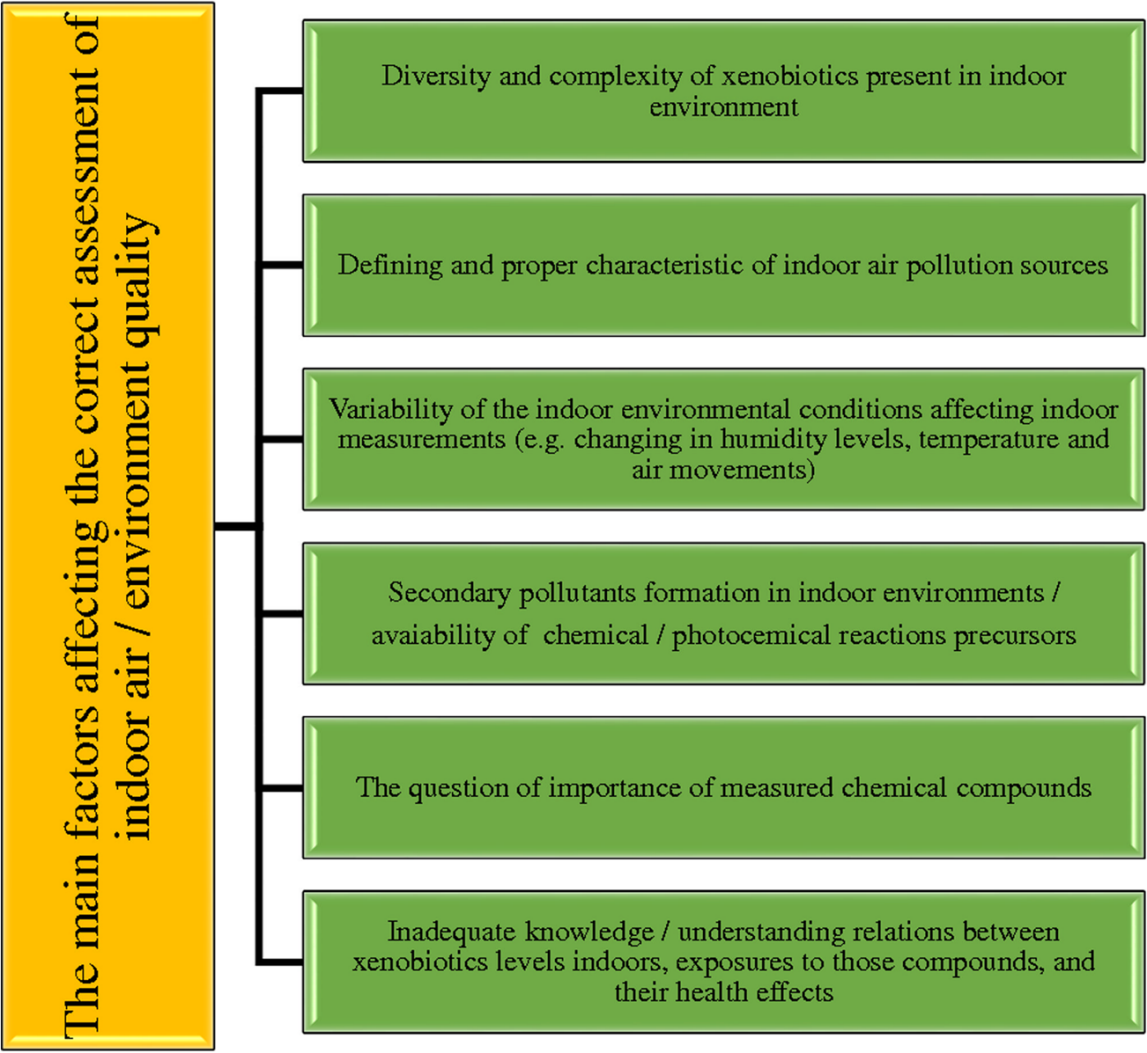
The main aspects that affect the difficulty to measure and estimate the indoor air quality (indoor environment) (based on the data published by Steinemann et al. (2017))

Although the presence of harmful chemical compounds in the indoor environment (such as benzene and formaldehyde—chemical compounds classified by experts from the International Agency for Research on Cancer (IARC) as group 1 carcinogens) has been measured in a lot of research conducted in this field, the type and the maximum allowable/permissible concentrations or amounts of harmful chemicals in indoor air are still not clearly defined by the appropriate legal regulations. In the vast majority of countries, such as the USA, the European Union countries, or Australia, only the type and the values of maximum allowable/permissible concentrations of chemical compounds that may be present in the atmospheric air (defined as “outdoor air” or “ambient air”) are clearly specified (Steinemann et al. 2017). The lack of clear and precise law regulations on the quality of indoor air in residential areas and public utilities is mainly caused by the difficulty in obtaining reliable analytical information on the concentrations of chemical compounds in the given indoor environment, and also by the variability and diversity of chemical compounds occurring in indoor air. Figure 1 schematically shows the main factors significantly affecting the evaluation of air quality in residential areas (Steinemann et al. 2017) and the impact of defined chemical compounds on human well-being and health.

Inorganic pollutants have also a very important meaning for shaping of the indoor air quality, especially in case of the residential rooms. Their presence in the indoor air is mainly related to human activities and to the exchange rate of air masses with atmospheric air. The main examples of indoor air inorganic pollutants are carbon monoxide, carbon dioxide, water vapor, tropospheric ozone, radon, or nitrogen oxides (Arbex et al. 2007; Hubbard et al. 2005; McCann et al. 2013; Szczurek et al. 2015; Baeza et al. 2018). Moreover, tropospheric ozone is the special case of inorganic indoor air pollutant due to the fact that it can take a part in reactions with various types of organic pollutants present in indoor environment. As a consequence, the formation process of secondary organic pollutants occurs. Such indoor environment reaction products might be more toxic and harmful for occupants of indoor areas than primary pollutions (Morrison and Nazaroff 2002; Aschmann et al. 2002; Nicolas et al. 2007). Due to this fact, the issue of the occurrence of inorganic pollutants, its reactions with organic compounds, and their impact on the human health is still a very interesting and important subject of studies many scientific and research centers.

Analyzing the literature data during the last two decades, it was noticed that several important national and international projects concerning the indoor air quality were developed. The primary aims of those research projects were (i) the evaluation of the air quality in such micro-environments like private homes (households and apartments), the workplaces, or public buildings (schools, kindergartens, etc.) and (ii) the estimation of the risk assessment caused by the harmful (carcinogenic) compounds measured in defined indoor areas. The correct assessment of human exposure to harmful compounds involves the necessity of combining the information about everyday lifestyle of inhabitants (in a case of private homes) or employees (in a case of workplaces) and the indoor air quality monitoring research—defined by the type and the amount of defined chemical compounds present in a gaseous phase of indoor micro-environment. The following scientific and social projects related to indoor air quality study have been conducted during the last two decades (Bruinen de Bruin et al. 2008; Kotzias et al. 2009; Geiss et al. 2011; Sarigiannis et al. 2011):

(i) EXPOLIS—Air Pollution Exposure Distributions of Adult Urban Populations;
(ii) GerES—German Environmental Surveys;
(iii) INGA—German study on Indoor Factors and Genetics in Asthma;
(iv) MACBETH—Monitoring of Atmospheric Concentration of Benzene in European Towns and Homes;
(v) PEOPLE—Population Exposure to Air Pollutants in Europe;
(vi) NHEXAS—National Human Exposure Assessment Survey;
(vii) AIRMEX—European Indoor Air Monitoring and Exposure Assessment;
(viii) INDEX—The Critical Appraisal of the Setting and Implementation of Indoor Exposure Limits in the EU.

In the mentioned developed scientific and social projects, obtained database contains the information about the air quality in residential homes considered as a single indoor area, without dividing it into separate individual specific indoor areas (as single indoor environment characterized by the specific type of destination and performed activities).

Despite many limitations and drawbacks that occur in the assessment of indoor environment quality, there is still a strong need to conduct monitoring studies aimed at obtaining reliable analytical data on the type and the amount of xenobiotics present in the air in residential areas (Marć 2017). Conducting monitoring research on indoor air quality with a specific intended use, exposure time, and equipment applied, there seems to be particularly interesting. These types of specific indoor areas include (i) areas for cooking and dining—private and commercial kitchens, (ii) private garages attached to residences or large underground car parks, and (iii) indoor areas intended mainly for the food storage and various types of preserves—basements located directly under the household. The harmful chemical compounds which occur in the air of these specific indoor areas might migrate to other residential spaces—more frequently used areas like bedrooms or living rooms, affecting the deterioration of the air quality in these areas.

The paper summarizes the literature data on indoor environment quality (mainly defined by the type and the amount of the organic chemical compounds present in it) in selected specific indoor spaces which contain an integral part of the residential area, such as kitchens, garages, and basements. According to the information which might be received from Scopus website database, the number of papers that concerns air quality in various types of kitchen areas (only research articles) is nearly 260. As for the other everyday use spaces dedicated to specific purposes, described in the following paper, the number of research articles concerns indoor air quality in basements is approximately 50, and in garages and car parks is approximately 80. Taking into account this screening information, it can be noticed that number of research articles in which the indoor air quality in kitchen areas is the main issue is approximately five times greater than in a case of research articles that concern the air quality in basements and more than three times greater than in a case of research articles that concern the quality in garages/car parks.

Conducting studies on the quality of the indoor environment in these specific enclosed areas allow the illustration of the impact of human activity (its daily activities and habits, hobbies, etc.) on a type and the amount of organic chemical compounds introduced into indoor air. In the described specific indoor spaces of residential areas, the appearance of human activity associated with the routine or/and spontaneous activities performed in them has an important impact on indoor air quality. The issue of presented paper is focused only on the occurrence and health impact of organic pollutants which might be present in mentioned specific indoor environments.

## Air quality in various types of food cooking areas/places

Considering the available literature data, one could be noticed that the quality of the indoor air in places where food is prepared and eaten is still an interesting research area. Generally, indoor areas designed and used for preparing meals can be divided into three main groups (Vainiotalo and Matveinen 1993; Raman et al. 2009; Hasan et al. 2009; Li et al. 2012; Pokhrel et al. 2015):

(i) Kitchens situated in individual flats or households where the cooking/meal preparation process takes place mostly using electric or gas cookers (the fuel is natural gas supplied from special containers or directly from the municipal line);
(ii) Indoor areas intended for meal preparation during the day where the cooking process is conducted mostly using low-efficiency cookers or stoves (open fire or leaky stoves) and where the source of heat (cooking fuel) is the process of burning solid fuels mostly biomass, crop residues, wood, or low-quality coal;
(iii) Large-surface commercial kitchen areas (restaurant kitchens, school, or university kitchens) that are people’s workplace, where the process of meal preparation, cleaning, and maintaining dishes occurs on a continuous basis.

Kitchen areas where the food preparation process is conducted regularly are characterized by a specific micro-climate. This results from, among other things, the character of activities performed in such indoor areas, higher temperature, and humidity occurring in them (Begum et al. 2009). In indoor areas intended for daily meal preparation, an adult person spends a considerable percentage (over 10%) of his/her time during the day (after the bedroom, which is the place where a person spends the most time on a daily basis). This time does not only include the meal preparation process but sometimes also the eating and the cleaning after the meal.

The method of meal preparation, the cooking style, the kind of the cooking tools used, the temperature of the cooking process, the cubic capacity of the room, the efficiency of the ventilation system in the room, and the number of persons using defined indoor area influence significantly on the type and amount of chemical compounds present in the indoor environment. Additional factors that influence the concentration and type/class of chemical compounds in the air of kitchens are (i) the type of the meal being prepared (deep-fried potatoes or meat or steam-cooked vegetables, fish, and meat), (ii) the quality of products used, and (iii) conditions related to the culture/customs/traditions (Baumgartner et al. 2011; Huboyo et al. 2011; Molloy et al. 2012; Singh et al. 2014; Torkmahalleh et al. 2017).

Depending on the characteristics of the kitchen, the main source of emissions of the chemical compounds is the process of fuel combustion used for meal preparation in the cooking process or the process of meal preparation itself (Kuo et al. 2006).

### Indoor air quality in rural kitchens using solid fuels for the cooking process

According to the scientific literature information, nearly three billion people all over the world prepare their meals in kitchens on open fireplaces or using very simple stoves characterized by low efficiency. In such design solutions, the fuel that is the most frequently used in the cooking process (and sometimes also for heating the room) is a solid fuel defined as biomass (wood, crop residues, dung), charcoal, and low-quality coal. Very often, indoor areas where such stoves are situated are not equipped with a ventilation system or a fume extraction system. Thus, fumes generated during the biomass burning process are released directly into the place where the meal is being prepared (Rehman et al. 2011; Singh et al. 2014; Bensch et al. 2015). Moreover, according to the literature data, approximately 533 million of cooking activities using solid fuels are performed outdoors, especially in tropical regions. Due to this fact, there is a strong possibility that cooking fumes and emitted harmful pollutants impact the health quality not only on cooks but also on the other village members. Open fire cooking activities which occur outdoor might also have important impact on the quality of atmospheric air on a defined area (Edwards et al. 2017).

In poorly and medium-developed countries, it is women who are usually responsible for preparing meals in kitchens. In this way, they are the most exposed to the harmful influence of pollutants generated during the biomass burning process and the cooking process. Children are also exposed excessively to harmful chemical substances emitted together with fumes as they often accompany their mothers during the meal preparation (Bruce et al. 2004; Jerneck and Olsson 2013). According to information in the literature, the exposure of children to harmful fumes generated from the fireplace causes an increase in the number of children suffering from acute lower respiratory infections (ALRIs) and pneumonia (Bates et al. 2013). The presence of harmful compounds in the air in indoor areas used for meal preparation is caused by the incomplete combustion of biomass at fireplaces (open fire or stoves) and/or the burning process taking place with insufficient supply of air/oxygen (Saud et al. 2013). To a lesser extent, the air quality is influenced by the meal cooking process. Additionally, in poorly and medium-developed countries, an inadequate level of kitchen ventilation causes high concentrations of xenobiotics in indoor air, which constitutes a greater hazard for people staying there (Pokhrel et al. 2015). The literature data summarized in Table 1 allows to conclude that the biomass burning process is a significant source of emissions of harmful agents and chemical compounds. According to the information present in Table 1, inorganic compounds—CO, CO_2_, and trace elements, as well as some organic compounds mostly from the group of PAHs with proven carcinogenic properties, are the main chemical pollutants released into indoor air in kitchens.

**Table 1:** Information about chemical pollutants determined in the kitchen environments equipped with cooking stoves with different cooking fuels

Analytes in kitchen environment	Research area/subject	Fuel used in cooking stove	Average concentration level (or range)		Ref.
Trace metals: lead, iron, cadmium calcium, potassium, magnesium	Black solid materials deposited from biomass burning at the cooking stoves in Narsingdi, Dhaka, Bangladesh	Rice, husk coils	Pb—95.6 mg/kg, Fe—11,520 mg/kg, Cd—8.33 mg/kg, Ca—1635 mg/kg, K—17.1 mg/kg, Mg—443.1 mg/kg		Hasan et al. (2009)
		Mixed (straw, bamboo, cow dung, leaves, and plants)	Pb—125.2 mg/kg, Fe—12,360 mg/kg, Cd—12.0 mg/kg, Ca—1648 mg/kg, K—21.5 mg/kg, Mg—534.2 mg/kg		
Black carbon	Solid biomass-based cooking in traditional mud stoves in northern India	Wood burning	Morning cooking: 54 ± 73 μg/m^3^ (range from 3 to 1970 μg/m^3^); evening cooking: 62 ± 61 μg/m^3^ (range from 3 to 1070 μg/m^3^)		Rehman et al. (2011)
PM_2.5_	Household kitchens of Bhaktapur, Nepal	Kerosene	169 ± 207 μg/m^3^		Pokhrel et al. (2015)
		Rice husk	759 ± 988 μg/m^3^		
		Biomass (wood and rice husk)	656 ± 924 μg/m^3^		
		Wood	630 ± 908 μg/m^3^		
PM_10_ and CO	Nouna, Kossi, northwest Burkina Faso	Wood	PM_10_—2553 μg/m^3^ (range from 1811 to 3295 μg/m^3^); CO—17.0 ppm (range from 12.4 to 21.5 ppm)		Yamamoto et al. (2014)
		Charcoal	PM_10_—2559 μg/m^3^ (range from 1646 to 3472 μg/m^3^); CO—16.8 ppm (range from 11.7 to 21.8 ppm)		
PM_2.5_, TSP	Rural Purepecha community in Michoacan, Mexico	Wood burned in open fire stove	PM_2.5_—0.257 ± 0.176 mg/m^3^; TSP—0.317 ± 0.188 mg/m^3^		Armendáriz-Arnez et al. (2010)
		Wood burned in improved Patsari stove	PM_2.5_—0.101 ± 0.052 mg/m^3^; TSP—0.143 ± 0.065 mg/m^3^		
PAHs (benz(a)anthracene, benzo(k)fluoranthene, benzo(b)fluoranthene, benzo(a)pyrene, di-benz(a,h)anthracene, indeno(1,2,3-cd)pyrene, chrysene)	Village Mall, 40 km north-west of Lucknow City, India	Cow dung cake	ΣPAHs in summer season—16.12 ± 5.33 μg/m^3^ (range from 2.23 to 46.07 μg/m^3^); ΣPAHs in winter season—33.35 ± 8.70 μg/m^3^ (range from 5.72 to 70.67 μg/m^3^)		Bhargava et al. (2004)
		Wood	ΣPAHs in summer season—9.11 ± 3.57 μg/m^3^ (range from 1.93 to 40.46 μg/m^3^); ΣPAHs in winter season—15.63 ± 2.95 μg/m^3^ (range from 4.34 to 26.81 μg/m^3^)		
22 pPAHs	A rural non-smoking household in Zhuanghu, northern China	Biomass burned in traditional Chinese rural cook stove	ΣpPAHs in winter season—6.1 ± 3.1 μg/m^3^; ΣpPAHs in summer season—2.4 ± 1.6 μg/m^3^		Ding et al. (2012)
10 PAHs, 11 nitro derivatives of PAHs (NPAHs)	Rural area of Pong Yeang, Mae Rim District, Chiang Mai Province, in the northern part of Thailand	Open stoves fueled by wood	ΣPAHs in dry season—range from 3.54 to 9.99 μg/m^3^ (mean 6.50 μg/m^3^); ΣNPAHs in dry season—range from 0.011 to 0.019 μg/m^3^ (mean 0.015 μg/m^3^)		Orakij et al. (2017)
Nitrated PAHs, oxygenated PAHs	Rural area of Heshun County, in Shanxi Province, China, during a non-heating period; randomly selected four villages: Songyuan, Yixing, Liyang, and Pingsong	Honeycomb briquette	ΣnPAHs—0.57–0.93 ng/m^3^; ΣoPAHs—14–18 ng/m^3^	Total concentrations of nPAHs—2.2 ± 2.5 ng/m^3^; total concentrations 1of oPAHs—230 ± 520 ng/m^3^	Chen et al. (2017a)
		Wood	ΣnPAHs—4.5 ng/m^3^		
		Peat (cake of coal–clay mixture)	ΣnPAHs—3.9 ng/m^3^; ΣoPAHs—74 ng/m^3^		
PM_2.5_, CO	Kopiwatta, a rural community outside of Kandy, Sri Lanka	Traditional biomass cookstoves	PM_2.5_—369 μg/m^3^ (range from 97 to 940 μg/m^3^); CO—3.74 ppm (range from 0.74 to 8.66 ppm)		Chartier et al. (2017)
		Biomass burned in Anagi stoves (indigenous cookstove made from crushed fired clay and fillers produced locally)	PM_2.5_—218 μg/m^3^ (range from 86 to 471 μg/m^3^); CO—3.04 ppm (range from 0.82 to 6.90 ppm)		
PM_2.5_, CO	District Fatehgarh Sahib located in south-eastern part of Punjab State; four randomly selected villages: Bagh Sikander, Dubhali, Gopalon, and Khera	Solid biomass fuel (SBF)	PM_2.5_—8-h time-weighted average concentration—1526 μg/m^3^ (range from 1250 to 1860 μg/m^3^); CO—8-h time-weighted average concentration—13.13		Sidhu et al. (2017)
PM_2.5_, CO	Two villages in the Nyando Division of Nyanza Province in rural western Kenya	Biomass burned in traditional cookstoves (TCSs)—three-stone fire	PM_2.5_—geometric mean of the 48-h concentration—586 μg/m^3^ (range from 460 to 747 μg/m^3^); CO—geometric mean of the 48-h concentration—6.5 ppm (range from 4.9 to 8.5 ppm)		Yip et al. (2017)
		Six improved biomass cookstoves (ICSs)	PM_2.5_—geometric mean of the 48-h concentration—409 μg/m^3^ (range from 363 to 460 μg/m^3^); CO—geometric mean of the 48-h concentration—4.9 ppm (range from 4.3 to 5.5 ppm)		

For PAHs, their sources of emissions into the air in kitchens can be classified in two main groups (Moret and Conte 2000; Zhu and Wang 2003):

(i) Heating of cooking oil, in which PAHs occur as pollutants and are released together with the gaseous phase (vapors) from the oil surface;
(ii) As a result of high temperatures of the food frying process, unstable smaller particles (pyrolysis) are formed, which are then transformed into more chemically stable compounds from the PAHs (pyrosynthesis).

Information about the type and the amount of chemical compounds as well as solid particles emitted into the kitchen air make it possible to conclude that the process of heat generation (the process of burning biomass, coal of charcoal) has a greater influence on the air quality than the cooking process (frying, roasting, or stewing). Furthermore, chemical compounds released as a result of using an open fireplace in the kitchen influence not only the air quality but also, indirectly, the users of these indoor areas. Furthermore, they might be considered as a specific (uncontrolled) sources of emissions of chemical compounds into the atmospheric air (Moret and Conte 2000; Jeuland et al. 2015). Considering the design and construction aspects of kitchens in poorly and medium-developed countries, the occurrence of extreme building solutions can be noted (Salam et al. 2013):

- Kitchens with open fireplaces are completely enclosed or have a very small window to heat the entire indoor area as much as possible;
- Kitchens do not have an entrance door or three walls (a single wall that protects from the wind), and as a result, air circulation (in this case, circulation of atmospheric air) is very high at the place where meals are prepared and open fireplaces are used.

### The quality of air in household kitchens located in regular apartments or dwellings

In developed countries, in a large majority of cases, stoves/cookers are used for meal preparation where liquefied petroleum or natural gas is the source of heat (the quality of this fuel determines the type of chemical compounds released into the air) (Abdullahi et al. 2013). In this way, hazards related to emissions of pollutants into the kitchen air, connected with the burning process of solid fuels such as biomass, charcoal, or low-quality coal, have been significantly minimized (Huboyo et al. 2011). Moreover, in accordance with a new type of building philosophy, new flats and apartments do not have a gas pipeline connection to minimize the risk of a potential explosion. As a result, electric cookers are more often used for meal preparation in kitchens (Jeuland et al. 2015). Moreover, a ventilation system installed directly over the cooker/stove is an indispensable element in every kitchen to minimize the transport of pollutants and odors to adjacent rooms.

With regard to kitchens in developed countries, the type and amount of chemical compounds present in the indoor environment are mostly influenced by the meal preparation technique (frying: deep frying, stir frying, pan frying, shallow frying; roasting; toasting; grilling; boiling and broiling), cultural styles of cooking (Chinese, Western, fast food, African, Indian, Malay, etc.), the cubic capacity of the kitchen, the number of persons living in the apartments (a larger number of people determines the preparation of a greater number of meals; thus, the time of meal preparation and the time spent in the kitchen is longer), the frequency of meal preparation, and the efficiency of the ventilation system (Zhao et al. 2007; Li et al. 2012).

As a result of using the kitchen, pollutants can be released into the indoor air that affects human health. These includes fine particulate matter PM_2.5_ and PM_10_, PAHs, VOCs, saturated and unsaturated fatty acids, dicarboxylic acids, or n-alkanes (Buonanno et al. 2009; Hecht et al. 2010; Kim et al. 2011). Detailed information about the levels of chemical compounds released during various types of meal preparation processes in kitchens was described in the review article by Abdullahi et al. (2013). The authors described the influence of the meal preparation technique in kitchens (boiling, frying, or broiling) on the type and amount of chemical compounds released into the kitchen environment. Exhaustive information from the literature was also presented on the broad spectrum of analytical techniques used for sample collection (passive and dynamic sampling techniques), extraction techniques applied for analyte isolation and/or preconcentration (Soxhlet extraction, microwave extraction), and the tools and techniques used for the separation and quantitative determination of the chemical compounds (GC/MS or HPLC systems) released during the cooking process (Abdullahi et al. 2013).

On the other hand, in a concise manner, Kim et al. (2011) presented information on the effects of the type of oil usage (together with the temperature of the cooking process) during meal preparation on the type and amount of chemical compound emitted into the kitchen air. It was noticed that aldehydes are released into the indoor air during the cooking process (mean values were from 80.4 to 3869 mg/h/L) (Kim et al. 2011).

To improve the air quality in kitchens, some model tests are conducted under laboratory conditions. These types of studies are aimed at determining the effect of the type of the meal prepared and the heating device used, on the kitchen air quality. Taking into account the literature information, such model solutions were proposed by the following:

(i) L’Orange et al. (2012), where the authors determined the amount of suspended particle/matter PM_10_ with the use of various types of cookers and different cooking temperatures (L’Orange et al. 2012);
(ii) Gao et al. (2015), where research was performed using a kitchen chamber, which was specially designed and constructed for this purpose, together with a system of measuring devices to determine the type and the amount of PAHs released during the cooking process (Gao et al. 2015).

### The quality of air in large commercial kitchens

Another type of indoor areas used for meal preparation is multi-station commercial kitchen in restaurants, at campuses, hospitals, schools, etc. The specification of kitchens is associated with three general factors (Kuo et al. 2006; Singh et al. 2016):

(i) This location is a workplace for a specific number of people (professional cooks, waiters), and for this reason, the time they spend in these indoor areas is much longer than for the average person, especially since the working hours are often irregular. Due to a very long exposure time (even up to 60% during the day), commercial employees in multi-station kitchens are considerably exposed to the harmful effects of compounds released during the meal preparation process;
(ii) A very large number of stations intended for meal preparation are situated very close to one another in a small space, and a large number of people work at these stations. Additionally, many more meals are prepared in commercial kitchens than in individual household kitchens;
(iii) Kitchens are usually located on the lowest floors of the building, which results in a very small number of windows and a permanently elevated temperature. Due to this fact, it is necessary to install a central ventilation system to remove gaseous pollutants formed during the cooking and meal preparation process.

The presence of a large number of devices for meal preparation (grills, ovens, hot plates, fryers, kettles, pasta boiler, etc.) in commercial kitchens causes a significant increase of the temperatures and higher nuisance of working conditions for persons who work there. An increase in the temperature could also influence a potential increase in the emissions of chemical compounds into the kitchen indoor air. Working conditions in multi-station commercial kitchens were compared in the Matsuzuki’s study (large-scale kitchen in schools and hospitals; small-scale kitchen in pubs and family restaurants) in terms of the air temperature, the radiant heat index value, and the wet bulb globe thermometer (WBGT) index (defined in °C). It was shown that the type of the cooker used has a significant influence on the air temperature in such indoor area. If electric cookers are used, the air temperature inside the building observed during the research was higher than for gas cookers (Matsuzuki et al. 2011).

The aforementioned environmental factors, such as humidity, radiant heat, and airflow, significantly influence the comfort of work and well-being of employees at multi-station commercial kitchens. The type and amount of chemical compounds released into the indoor air during the meal preparation process is a significant factor that affects health of people working in commercial kitchens (Saha et al. 2012). Due to considerably higher numbers of prepared meals and a higher number of potential sources of pollutant emissions into the indoor environment than in the case of household kitchens, xenobiotic concentrations in the air of commercial kitchens can be very high and cause a valid threat to users’ health (Chen et al. 2012).

Table 2 presents and summarizes the literature data on the results of research on the quality of air in the various types of commercial kitchens. According to the data presented in Table 2, main groups of environmental pollutants in commercial kitchens that occur as a result of meal preparations are PAHs, CO, CO_2_, and suspended matter of PM_2.5_ and PM_10_.

**Table 2:** Information about the air pollutants measured in indoor air in various types of commercial kitchens

Measuring analytes	Place of conducting research	The most commonly applied cooking method	Sampling technique	Final determination technique	Average concentration level (or range)	Ref.
12 PAHs (naphthalene, acenaphthene, fluorene, phenanthrene, anthracene, fluoranthene, pyrene, benzo[a]anthracene, chrysene, benzo[e]-pyrene, benzo[k]fluoranthrene, benzo[a]-pyrene)	Four commercial kitchens located in hotels in Hangzhou (China)	Boiling, frying, and broiling in a pan	Dynamic sampling system with Whatman glass fiber filter (GFF, 25 mm, Whatman, England) and XAD-2 (2.5 g)	HPLC system (Hitachi, L-7000 series, Japan) with a fluorescence detector (Hitachi, L-7480, Japan)	ΣPAHs were ranged from 10 to 21 μg/m^3^	Zhu and Wang (2003)
CO_2_	Four Chinese commercial restaurants located in Xi’an metropolitan area	Fried, stewed, or braised	–	Indoor air quality analyzer TSI-7545 (range from 1 to 5000 ppm)	1 m within the cooking range—from 586 to 2145 ppm, 3 m wide, 1 m away from cooking range—from 546 to 956 ppm	Li et al. (2012)
VOCs (n-heptane, ethyl acetate, nonanal, n-octane, and toluene)	A university canteen that serves the school of architecture (Turkey)	Deep-frying palm oil margarine	Tenax TA in stainless steel thermal desorption tubes filled with 100 mg sorbent (SKC 226–340)	Gas chromatography (Agilent 6890N) coupled with mass spectrometry (Agilent 5973Nms) system	n-heptane—83.0 μg/m^3^, ethyl acetate—24.9 μg/m^3^, nonanal—23.4 μg/m^3^, n-octane—16.6 μg/m^3^, toluene—4.4 μg/m^3^	Sofuoglu et al. (2015)
Aldehydes (hexaldehyde, acetaldehyde, formaldehyde)			DNPH-coated silica gel sorbent tubes with a 300-mg front sorbent and a 150-mg backup sorbent (SKC 226–119)	Agilent 1100 Series high-performance liquid chromatography coupled with an ultraviolet visible absorption detector operated at 360 nm	Hexaldehyde—1.29 μg/m^3^, acetaldehyde—13.1 μg/m^3^, formaldehyde—2.95 μg/m^3^	
PM_10_			3M Quest EVM-7	90° optical light-emitting photometer	From 279 to 1583 μg/m^3^	
PM_2.5_			37-mm glass fiber filters using a Harvard impactor coupled with a sampling pump (SP 280E; Air Diagnostics and Engineering Inc.)	Weighing on a precision balance with a 10-μg resolution (Sartorius CPA 225D) before and after sampling	From 76 to 158 μg/m^3^	
CO	Four kitchens in the large campus in India	Boiling and frying	–	Indoor air quality measurement device—IAQ Calc7545	From 350 to 1710 ppm	Saha et al. (2012)
CO_2_					From <1 to 102.1 ppm	
22 PAHs (naphthalene, acenaphthylene, acenaphthene, fluorene, phenanthrene, anthracene, fluoranthene, pyrene, benzo(c)-phenanthrene, benzo(b)napth(2,1-d)thiophene, cyclopenta(cd)pyrene, benz(a), anthracene, chrysene, benzo(b)fluoranthene, benzo(k)fluoranthene, benzo(e)pyrene, benzo(a)-pyrene, indeno(1,2,3-d)pyrene, dibenz(ah)anthrancene,benzo(ghi)perylene, anthanthrene, coronene)	Three types of popular vendors from the night markets of Taichung City, Taiwan	Grilling food (grill powered by charcoal fuel or electricity)	The personal air collection samplers (SKC model, 224-PCXR8) with personal environmental monitors (10-mm PEM, SKC model 200), the quartz filters (SKC high-purity quartz filter, 37 mm, binder-free)	Gas chromatography with a flame ionization detector (PerkinElmer Auto-system, model N611–9000)	ΣPAHs were ranged from 1.69 to 31.0 μg/m^3^ (charcoal fuel); ΣPAHs were ranged from 0.51 to 0.73 μg/m^3^ (electricity)	Kuo et al. (2006)
PM_10_				–	From 1.49 to 17.2 mg/m^3^ (charcoal fuel); from 0.55 to 1.5 mg/m^3^ (electricity)	
18 carbonyl compounds in C_1_–C_10_ range	Six restaurants located in urban Kaohsiung, Taiwan	Grilling, roasting, boiling, baking, and frying	Silica cartridge impregnated with 2,4-dinitrophenylhydrazine	High-performance liquid chromatography (HP-1100, Agilent Technologies, USA)	Range from 8.59 to 45.48 ppb in kitchen area; range from 58.02 to 132.10 ppb in exhaust streams	Cheng et al. (2016)

Due to the fact that a broad spectrum of harmful substances can be emitted, during the meal preparation process, the terms “cooking fumes (CFs) or cooking oil fumes (COFs)” were introduced into scientific literature. These terms defines both suspended matter (PM_10_, PM_2.5_) and chemical compounds (inorganic and organic compounds) emissions (Svedahl et al. 2009), apart from the fact that, in accordance with information presented by experts from the International Agency for Research on Cancer (2010), substances formed during meal preparation were classified as a “probable human carcinogen—group 2A” (IARC 2010). Furthermore, according to the data published by Wei et al. (2017), the exposure to cooking oil fumes emitted during cooking process in household kitchens might have direct impact on the poor sleep quality among residents of defined indoor area (Wei et al. 2017).

## Air quality in garages attached to residences and large-scale underground car parks

Another example of a specific micro-environment in terms of the composition (both quantitative and qualitative) and the time spent by adult men during the day are garage areas intended for storage of mechanical vehicles. Such indoor areas are defined as semi-enclosed structures and can be divided into two main categories (Papakonstantinou et al. 2003; Demir 2015; Marć et al. 2016):

(i) Residential/attached vehicle garages, where the space for the mechanical vehicle is directly attached to the residential section or a household;
(ii) Large-surface and/or multi-staged underground car parks, where a lot of parking spaces have been designated on a defined surface area. These indoor areas are intended for employees of corporations, large companies, clients of large-surface shopping centers, or inhabitants of high-rise buildings and apartment buildings.

The specificity of air quality in garages is conditioned by the fact that the average person spends very little time in such indoor areas (a few or several minutes) during the entire day. However, the concentration of harmful chemical substances in the indoor air of garages could reach a very high level (Batterman et al. 2006). For this reason, despite a very short time spent by people in such spaces, the high level of harmful chemical compounds contained in the indoor air can significantly affect the functions of the human body, causing headaches, dizziness, mucous membrane irritation, or induce allergic reactions (Edokpolo et al. 2014; Moolla et al. 2015). For this reason, the air exchange system plays an important role in the formation of indoor air quality. Effective ventilation allows controlling air quality by diluting and displacing of indoor contaminants. Introducing the ambient air into the indoor areas may help to achieve desired indoor comfort. Although, it should be pointed that the perceived comfort depends on individual human preferences.

A significant problem, from the point of view of users of all kinds of indoor areas, is the process of pollutant migration which is present in the garage environment direct to the indoor air of other small household rooms. While analyzing the air quality in garage areas directly adjacent to residential rooms, it should be remembered that they are not intended to be only the parking spaces for mechanical vehicles. They are also a storage places for their users where various types of gasoline storage containers, solvents, oils, paints, building materials, etc., are kept (Hun et al. 2011; Marć et al. 2016). Sometimes, such spaces are also the workshop where various repair and maintenance works are performed. This activity increases the levels of organic compounds in the indoor air, which, on the other hand, extends the exposure time, and this can have negative consequences for the health in a longer perspective (Marć et al. 2016). With regard to the information published in the literature, which concerns air quality in garages that are directly adjacent to other residential places, it was observed that VOCs (mostly compounds from the BTEX group and alkylbenzenes), monoterpenes, and CO are the main group of compounds determined in the air. Table 3 presents the literature information about the levels of main chemical compounds determined in the garage air directly adjacent to residential areas.

**Table 3:** Information about analytes determined in indoor air in several residential attached garages

Analyte determined in garage indoor air	Localization	Sampling technique	Final determination technique	Average concentration level (or range)	Ref.
Benzene	Residence with an attached garage, San Antonio, USA	• 75-μm film solid phase micro-extraction carboxen/polydimethylsiloxane (SPME-CAR/PDMS) fiber (Supelco) • Passivated and pressurized whole-air canister samplers	GC-FID	2.3 ± 2.4 ppbv	Zielińska et al. (2012)
Toluene				5.4 ± 5.3 ppbv	
Ethylbenzene				0.9 ± 0.8 ppbv	
m/p-Xylene				3.1 ± 2.6 ppbv	
o-Xylene				1.2 ± 0.9 ppbv	
1,3-Butadiene				0.1 ± 0.2 ppbv	
MTBE				1.1 ± 1.0 ppbv	
Formaldehyde		Acidified 2,4-dinitrophenylhydrazine cartridges	HPLC-DAD	7.7 ± 1.5 ppbv	
Acetaldehyde				2.6 ± 1.5 ppbv	
CO		Passivated and pressurized whole-air canister samplers	Converted to methane for analysis by GC-FID	0.8 ± 0.7 ppm	
Benzene	Residential garages to adjoining houses in 15 homes in southeast Michigan, USA	Passive tube-type samplers and Tenax GR adsorbents	GC-MS	36.6 ± 38.5 μg/m^3^	Batterman et al. (2007)
Toluene				214.3 ± 180.3 μg/m^3^	
Ethylbenzene				28.0 ± 23.7 μg/m^3^	
m,p-Xylene				114.0 ± 97.0 μg/m^3^	
o-Xylene				38.0 ± 32.7 μg/m^3^	
α-Pinene				6.8 ± 10.7 μg/m^3^	
d-Limonene				6.5 ± 5.2 μg/m^3^	
Naphthalene				8.9 ± 8.7 μg/m^3^	
1,2,3-Trimethyl benzene				10.3 ± 8.8 μg/m^3^	
1,2,4-Trimethylbenzene				44.0 ± 37.6 μg/m^3^	
1,3,5-Trimethylbenzene				12.4 ± 10.3 μg/m^3^	
Benzene	A garage containing two cars attached to the house located in Ann Arbor, MI, USA	Stainless steel sampling tube filled with a Tenax GR adsorbent	GC-MS	19.0 μg/m^3^ ± 4.6%	Batterman et al. (2006)
Toluene				114.8 μg/m^3^ ± 3.9%	
p-Xylene, m-xylene				65.8 μg/m^3^ ± 6.8%	
1,2,4-Trimethylbenzene				19.7 μg/m^3^ ± 10.4%	
Naphthalene				3.4 μg/m^3^ ± 10.1%	
α-Pinene				0.9 μg/m^3^ ± 5.7%	
d-Limonene				7.9 μg/m^3^ ± 10.2%	
Methylene chloride	Attached garages of residences in the Boston, MA, USA	Active sample collection with the use of a custom-made triple-bed thermal desorption tube with 200 mg of Carbopack B, 230 mg of Carbopack X, and 170 mg of Carboxen 1001 (Supelco/PerkinElmer, Bellefonte, PA)	GC-MS	9.8 ± 36 μg/m^3^	Dodson et al. (2008)
Chloroform				0.08 ± 0.08 μg/m^3^	
Trichloroethene				3.3 ± 10 μg/m^3^	
Tetrachloroethene				2.8 ± 7.8 μg/m^3^	
1,4-Dichlorobenzene				2.3 ± 8.4 μg/m^3^	
1,3-Butadiene				7.4 ± 18 μg/m^3^	
MTBE				131 ± 338 μg/m^3^	
Benzene				58 ± 145 μg/m^3^	
Toluene				102 ± 69 μg/m^3^	
Ethylbenzene				35 ± 39 μg/m^3^	
m,p-Xylene				90 ± 64 μg/m^3^	
o-Xylene				35 ± 36 μg/m^3^	
Styrene				3.4 ± 5.3 μg/m^3^	
α-Pinene				38 ± 110 μg/m^3^	
d-Limonene				7.3 ± 13 μg/m^3^	
Formaldehyde		Active sampling using acidified 2,4-dinitrophenylhydrazine-coated silica cartridges (Waters Corp, Milford, MA)	HPLC-UV-VIS	6.7 ± 6.4 μg/m^3^	
Acetaldehyde				8.9 ± 8.6 μg/m^3^	
Benzene	Three types of residential garages in the Tri-City agglomeration (Gdańsk, Gdynia, Sopot), Poland	Radiello® diffusive passive samplers with graphitized charcoal cartridge as a sorption medium	GC-FID	Range from 5.9 to 53 μg/m^3^	Marć et al. (2016)
Toluene				Range from 7.1 to 195 μg/m^3^	
Ethylbenzene				Range from 3.0 to 39 μg/m^3^	
o-Xylene				Range from 5.6 to 44 μg/m^3^	
p,m-Xylene				Range from 6.3 to 99 μg/m^3^	

A very important and dangerous phenomenon is the fact that pollutants emitted and present in the air of garages adjacent to the residential building are transported to other indoor areas intended for the permanent stay of people, such as the kitchen, bedroom, or living room. Batterman et al. (2007) concluded in their research that the main cause of the presence of a broad spectrum of organic compounds, especially those with carcinogenic properties (such as benzene) in the indoor air of residential areas, is the fact of having an adjacent garage as a consequence and the migration of those compounds to the air of other residential places (Batterman et al. 2007). According to information published by Dodson et al. (2008), the direct vicinity of households with an attached garage causes a significant increase (even up to 40%) in the levels of compounds such as BTEX and methyl tert-butyl ether (MTBE). The origin of these compounds is directly connected with the activity and use of mechanical vehicles with internal combustion engines in which liquid fuel is used (Dodson et al. 2008).

Graham et al. (2004) conducted modeling studies using 31 compounds from the group of non-methane hydrocarbons (NMHCs) and showed that, depending on the temperature of engine operation (cold-start test or hot-soak test) of the mechanical vehicle in the garage, the transport of pollutants from the garage air to the air in indoors accounts for 9 up to even 85% of the content of chemical compounds in areas intended for permanent human residence (Graham et al. 2004).

Another type of indoor areas intended for temporary storage of mechanical vehicles are large-area underground car parks usually equipped with a central automated ventilation system (Papakonstantinou et al. 2003). In such semi-enclosed spaces (with limited access of fresh air), the influence on the type and quantity of chemical compounds present in the air of large-area underground car parks is mostly caused by the activity and characteristics of mechanical vehicles, i.e., the type of the fuel used, driving style, engine temperature, type of the engine oil used, and the number of mechanical vehicles left there (de Castro et al. 2015). Large-area car parks might also be considered as a potential source of emissions (defined as a “hot spot”) of the VOCs into the surrounding atmospheric air (Kim et al. 2007).

Users of underground car parks (usually employees of companies) spend only a very short time in them during the day (from a few to several minutes); however, considering the high content of xenobiotics in the indoor air and the regularity of their activity (daily work from Monday to Friday), the threat to people resulting from the presence of harmful chemical compounds at these places is very real (Kim et al. 2007). For this reason, the results of air quality monitoring tests obtained inside multi-store car parks are more and more often used, and the additional degree of human exposure to harmful chemical compounds present in the gaseous phase of a given car park is also estimated (Edokpolo et al. 2014).

Vukovic et al. (2014) in their research work characterized the air quality in a large-surface car park located in Belgrad, in terms of the content of PM_10_, major and trace elements, and PAHs. Using the obtained research results and generally available statistical data on time spend by the users, their age and weight, basic parameters defining the degree of human exposure to harmful chemical compounds—carcinogenic and non-carcinogenic health risk, exposure to carcinogenic compounds were determined (Vukovic et al. 2014).

Glorennec et al. (2008), on the other hand, using the data of the research related to studying concentrations of CO, H_2_S, NH_3_, NO_2_, SO_2_, PM_10_, PM_2.5_, 9 VOCs, 13 PAHs, 5 aldehydes and ketones, and 6 metals at the underground car park, made an attempt to estimate and assess the health hazard for users of the studied indoor environment (Glorennec et al. 2008). In routine monitoring tests aimed at obtaining analytical information on the quality of indoor air at underground car parks, the most frequently determined parameters include carbon monoxide concentrations (Papakonstantinou et al. 2003; Duci et al. 2004), VOCs (mainly BTEX) (Jo and Song 2001), and PM_2.5_ and PM_10_ (Li and Xiang 2013; Giechaskiel et al. 2014).

## The quality of air in basements

Basement areas (placed directly under the residential building or in its close neighborhood) are characterized by very specific quality of micro-environment which is created mainly by such factors as (i) presence of the stored items—basements are most commonly used to storage of the unused furniture and other equipment, preserves, lacquers, paints, and gasoline products; (ii) high relative humidity of the air; (iii) very limited air exchange; (iv) house dust presence; and (v) mold and mildew.

The above-mentioned factors affect the formation of the characteristic odor/smell which is so-called “basement smell.” The main reason of its formation is putrefaction processes in high humidity conditions and with the participation of micro-organisms. Under these conditions, molds and its spores arise and become biological air pollution in the basements.

The quality of air in the basements can affect the formation of health disorders (from headache and fatigue to cancer) due to large number of potential VOC emission sources, although generally, there is a temporary exposure (short term). According to the literature data, the main danger is the possibility of uncontrolled transport of the pollutants to air in residential rooms (when the basement is located directly under other rooms in the apartment).

Du et al. (2015) in their research work (Detroit, USA) demonstrated that the rate of air exchange in basements is higher in winter than in summer, which may affect the increased migration potential of VOCs to the living areas (Table 4). Moreover, authors concluded the presence of higher concentrations of benzene and toluene in the basement than in living areas—this can be caused due to storage of the organic solvents and gasoline-powered equipment in the basement. It has been proven that potential sources of emissions are located in 75% of the studied basements. The impact of these sources on the formation of air quality in residences, determined using the basement/indoor concentration ratios, is significant in many cases (*B*/*I* > 1). It was also noted that it is reasonable to use the instructions of storage of some solvents and items (older equipment which may leak and release toxic vapors, e.g., benzene) in the basement areas (Du et al. 2015).

**Table 4:** General information about chemical compounds determined in basement indoor environments

Localization	Determined compounds	Sampling technique	Applied sorption medium	Technique for the separation/liberation of analytes	Final determination technique	Average concentration	Ref.
2 residential basements^a^, Massena and Lisbon, NY, USA	Methanol	Dynamic (400-mL, 1-L, and 6-L evacuated stainless steel canisters)	Lack of applied sorption medium	Lack of applied analyte liberation technique—direct injection of gas sample	GC-FID	5839 ± 3800 ppb	Soto-Garcia et al. (2015)
	Pentane					100 ± 61 ppb	
	Pentanal					86 ± 53 ppb	
	Hexanal					214 ± 176 ppb	
74 residences, Detroit, MI, USA	VOCs	Passive	Not mentioned	Thermal desorption	GC-MS	Benzene: 5.88 μg/m^3^, toluene: 77.30 μg/m^3^, naphthalene: 65.78 μg/m^3^, limonene: 21.95 μg/m^3^	Du et al. (2015)
55 residences, Boston, MA, USA	Formaldehyde	Dynamic	2,4-Dinitro-phenylhydrazine-coated silica cartridges (Waters Corp.)	Ozone scrubbers containing potassium iodide	HPLC-UV(operating at 360 nm)	12 μg/m^3^	Dodson et al. (2008)
	Acetaldehyde					8.5 μg/m^3^	
	Other VOCs	Dynamic	Triple-bed tube with Carbopach B, Carbopack X, and Carboxen	Thermal desorption	GC-MS	Methylene chloride: 9.5 μg/m^3^, chloroform: 0.57 μg/m^3^, trichloroethene: 0.43 μg/m^3^, tetrachloroethene: 1.7 μg/m^3^, 1,4-dichlorobenzene: 1.3 μg/m^3^, 1,3-butadiene: 0.5 μg/m^3^, MTBE: 8.8 μg/m^3^, benzene: 3.2 μg/m^3^, toluene: 21 μg/m^3^, ethylbenzene: 4.1 μg/m^3^, m,p-xylene: 12 μg/m^3^, o-xylene: 4.2 μg/m^3^, styrene: 1.7 μg/m^3^, α-pinene: 11 μg/m^3^, d-limonene: 8.8 μg/m^3^	

^a^Basements dedicated to store large fabric bags with blended mixture pellets

Similar conclusions about the seasonal differences in the air exchange rate in the basements were drawn by Dodson et al. (2008). A much higher concentration in the air in the basement was observed in the summer for the compounds such as trichloroethene, tetrachloroethene, styrene, o-xylene, 1,4-dichlorobenzene, α-pinene, d-limonene, formaldehyde, and acetaldehyde. Lower levels in winter associated with a dilution effect which takes place in this season due to greater air exchange rate, whereas as a source of emissions, it indicated primarily gasoline-powered equipment (e.g., motorcycle, trimmers, and boat engine) and stored gasoline or synthetic oil containers. In addition, for each compound covered by the research, the average percentage contribution to the shaping of air quality in the residential area (percentage contribution to the indoor concentration from the outside and each compartment within home) was identified. In addition, for each identified compound, their average percentage contribution to the indoor air quality were calculated (percentage contribution to the indoor concentration from the outside air and for each room within studied home). The obtained values varied from 0% for 1,3-butadiene to 22% for m,p-xylene (Dodson et al. 2008).

Radon, being a relatively heavy and radioactive gas, is unique basement pollution because of close proximity to the soil, where this harmful element might penetrate directly into the building environment (Roulet 2001). According to model studies, the geometry of the building has a significant impact on the penetration of radon into its interior through the basement (Wang and Ward 2000). The appropriate solution which allows avoiding of the excessive activity of radon is the use of optimal and efficient ventilation system in the basements (Roulet 2001).

The air quality in basements is the topic which requires control on the way of research. Currently, scientific literature does not provide enough data on this very important issue. Due to the wide range of pollution sources (both biological and chemical) and specific micro-climate conditions (high humidity, lower than average temperature, and very often lack of optima ventilation) carrying out of accurate research in such indoor areas is very difficult. The results suggest that increased ventilation is a very important component in basements, and this solution might be sufficiently for dissipation of the pollutants present in the gaseous phase. In a case of residential buildings, the consensus in the field of air quality in other living quarters might be also the solution in which the location of basement is not directly under the building, but as a separate building.

## Summary

The air quality in the residential areas is one of the main issues that require continuous monitoring and action to improve the living conditions of its inhabitants. Potentially occurring contamination of indoor air in residential rooms, performing at high concentration levels, can cause adverse health effects for people staying there. A special case is the indoor areas with the specific air quality, which is affected by the following factors:

(i) The intended use of the room and thus the exercise of specific actions by the inhabitants, which are part of everyday life;
(ii) The presence of equipment elements and stored items whose type is determined by the specific purpose of the room;
(iii) The lifestyle of the inhabitants, their social status, and cultural factors;
(iv) The intensity of air exchange rate.

Improvement of indoor air quality in residential buildings requires the implementation of programs, including periodic monitoring of the air pollutants. In addition, there should be undertaken actions aiming at raising public awareness about the possibility of the occurrence of pollutant emissions from equipment elements placed in buildings and apartments. This would allow the consumers to make conscious choices during the designing and arrangement of the indoor areas—in order to reduce the possibility of occurrence of the equipment characterized by high pollutant emission rate.

An important issue is also the use of effective ventilation systems, and if this is not possible—ventilation through frequent airing of the indoor areas. These actions enable the transport of accumulated pollutants into the atmospheric air, which in turn reduce (dilute) their content in rooms designed to accommodate people. Due to the lack of law regulations or national directives concerning air quality in residential room (especially in a specific indoor areas in which the concentration of harmful chemical compounds might be at high level), it is important to continue and develop the indoor air quality monitoring research in order to expand the awareness of the users of residential buildings. Also, information about the air quality in residential buildings might turn the attention of defined national or regional institutions on this important issue and become a baseline to take an appropriate actions and steps (including public consultations) to create basic law regulations or national directives about the indoor air quality in residential buildings, apartments, or flats.
